# Effects of Transgenic Rice Infected with SRBSDV on Bt expression and the Ecological Fitness of Non-vector Brown Planthopper *Nilaparvata lugens*

**DOI:** 10.1038/s41598-017-02218-w

**Published:** 2017-07-24

**Authors:** Hong-Xing Xu, Xiao-Chan He, Xu-Song Zheng, Xiao-Jun Zhou, Yong-Jun Lin, Zhong-Xian Lu

**Affiliations:** 1 State Key Laboratory Breeding Base for Zhejiang Sustainable Pest and Disease Control, Institute of Plant Protection and Microbiology, Zhejiang Academy of Agriculture Sciences, Hangzhou, 310021 China; 2 Jinhua Academy of Agricultural Sciences, Jinhua, 321017 China; 3 National Key Laboratory of Crop Genetic Improvement and National Centre of Plant Gene Research (Wuhan), Huazhong Agricultural University, Wuhan, 430070 China

## Abstract

The susceptibility of rice lines, T1C-19, T2A-1, and MH63 to SRBSDV infection are similar and the contents of cry protein in T2A-1 and T1C-19 do not change significantly. The survival rates of BPH nymphs feeding on SRBSDV-infected T1C-19, Bt T2A-1, or MH63 rice plants were not significantly different. The developmental stages of female BPH fed on T1C-19 plants infected with SRBSDV were significantly shorter than those fed on uninfected rice, while the males showed no significant difference. The duration of BPH feeding on SRBSDV-infected T2A-1 and MH63 also showed no significant difference in comparison with the respective control groups. Longevities of BPH adults feeding on SRBSDV-infected T1C-19, T2A-1 or MH63 were also not significant. However, the longevity of male adult BPH feeding on un-infected MH63 was significantly reduced in comparison with that of adult males feeding on un-infected T1C-19 and T2A-1 rice. In addition, the different rice lines and the rice plants infected and uninfected with SRBSDV did not significantly affect the sex ratio, female body weight, longevity, fecundity, or egg hatchability of BPH. In general, transgenic Bt rice infected with SRBSDV had little effect on the ecological adaptability of BPH.

## Introduction

Cultivating and using insect-resistant rice varieties is an economical, safe and effective method of controlling insect pests. However conventional breeding techniques used to produce new insect-resistant varieties often takes a long time. In addition there is lacking of resistant germplasm for major insect pests, such as stem borers (*Chilo suppressalis*, *Tryporyza incertulas*) and rice leaf folders (*Cnaphalocrocis medinalis*). One promising method to breednew insect-resistant varieties is by using transgenic technology to insert exogenous insect resistance genes into the rice genome^1, 2^. Tu *et al*.^3^ conducted a large-scale field experiment on transgenic rice, Minghui 63 and its hybrid Bt Shanyou 63, with a cry1Ab/Ac fusion gene showed that no insecticide was needed for the entire growth period. Most transgenic Bt rice strains show high insecticidal activity against *Chilo suppressalis*, *Tryporyza incertulas*, and *Cnaphalocrocis medinalis* ^2, 4, 5^. Ecological risks, such as the effects of transgenic rice on non-target arthropods have received considerable attention. Bt rice fields and control fields showed no significant differences in functional group dominancesuggesting that Bt rice had no significant effect on arthropod communities^6^. Diffusion experiments of rice planthoppers and leafhoppers in Bt rice and control rice fields showed that they did not show preference for Bt rice and Bt rice did not cause populations of non-target pests to increase^7^. When brown planthopper (BPH), *Nilaparvata lugens*, were allowed to freely select their host rice, there were no significant differences in selection or oviposition preference or fecundity in the Bt rice area and control area^7^. Some studies however showed that damages by non-target pests to Bt rice can be exacerbated^8^. Jiao *et al*.^9^ reported that populations of *Chilo suppressalis* and *Cnaphalocrocis medinalis* decreased in transgenic Bt “Shanyou 63” rice fields, but rice planthoppers and leafhoppers became the main pests. The Southern rice black-streaked dwarf (SRBSDV) disease, first reported in 2001 in Yangxi County, Guangdong Provinceis transmitted by the white backed planthopper (WBPH), *Sogatella furcifera* was named in 2008^10, 11^. The developmental duration of WBPH nymphs carrying SRBSDV and those feeding on rice infected with the SRBSDV might increase^12^. WBPH feeding behavior on plants with SRBSDV was found to be different from those on control. The frequency of phloem sap ingestion by WBPH with SRBSDV increased significantly, however the total feeding duration did not increase. Viruliferous WBPH with SRBSDV was found to feed on the phloem more frequently than the control thus contributing to virus transmission^13^. SRBSDV rice plant volatiles might also change the host selection behavior of WBPH^14^. For the non-vector BPH feeding on SRBSDV-infected rice significantly decreased the survival rates of nymphs and shortened the life span of female adults, while the hatching rates of eggs were increased^15^.

SRBSDV infection generally occurs in rice fields in Southern China causing serious economic losses. In this paper we explored the ecological fitness of a non vector on Bt rice infected with SRBSDV.

## Results

### Changes in infection rates of transgenic rice lines T2A-1 and T1C-19 infected with SRBSDV

The survival rates of SRBSDV inoculated plants T2A-1 and Bt T1C-19, as well as control line MH63 ranged from 92.27–95.00%. Infection rates of SRBSDV were 81.62–83.49%, and there were no significant differences among the three tested lines (Table 1).

**Table 1:** Infection rate of transgenic rice with SRBSDV.

Rice	Survival rate of rice seedling (%)	Infection rate of rice plants (%)
T1C-19	92.27 ± 6.47 a	83.49 ± 7.59 a
T2A-1	92.50 ± 5.40 a	83.18 ± 7.42 a
MH63	95.00 ± 6.24 a	81.62 ± 6.03 a

### Changes in Cry protein content in transgenic rice lines T2A-1 and T1C-19 infected with SRBSDV

SRBSDV infection had no significant effects on the Cry protein content of transgenic Bt rice lines T2A-1 and T1C-19 (Table 2).

**Table 2:** Cry protein contents in SRBSDV-infected transgenic rice.

Treatment	T1C-19 (μg/g)	T2A-1 (μg/g)
SRBSDV-infected rice plants	1.82 ± 0.32	16.77 ± 1.07
Un-infected rice plants	1.56 ± 0.21	20.07 ± 1.20
*P*	0.565	0.096

### Effects of transgenic rice plants infected with SRBSDV on the survival rates and duration of BPH

No significant changes of nymph survival rate were observed on BPH feeding on SRBSDV-infected T1C-19, T2A-1, or MH63 plants. The duration of the female BPH feeding on SRBSDV-infected T1C-19 plants were significantly shorter than those on uninfected plants, while the males showed no significant difference. There were no significant differences in the duration of male and female BPH feeding on SRBSDV-infected T2A-1 or MH63 plants. Rice variety and SRBSDV infection status did not significantly affect the survival rate of BPH, and there was no significant interaction between rice variety and SRBSDV infection. Rice variety had a significant effect on the duration of female BPH, while SRBSDV infection had no significant effect on this parameter, and there was a significant interaction between rice variety and SRBSDV infection. Rice variety had a significant effect on the duration of male BPH, but SRBSDV infection did not, and there was no significant interaction between rice variety and SRBSDV infection (Tables 3 and 4).

**Table 3:** Effects of SRBSDV-infected Bt rice on the nymph survival rate and duration of BPH.

Rice	Nymph survival rate (%)		Nymph duration of the female (d)		Nymph duration of the male (d)	
	Un-infected rice plants	Infected by SRBSDV	Un-infected rice plants	Infected by SRBSDV	Un-infected rice plants	Infected by SRBSDV
T1C-19	86.55 ± 2.45aA	80.71 ± 3.98aA	14.29 ± 0.23aA	13.52 ± 0.13bAB	13.58 ± 0.17aA	13.26 ± 0.18aA
T2A-1	83.51 ± 3.53aA	82.00 ± 5.83aA	13.77 ± 0.16aA	13.94 ± 0.28aA	12.78 ± 0.19aB	12.98 ± 0.27aA
MH63	79.91 ± 2.44aA	80.89 ± 3.02aA	12.69 ± 0.17aB	13.01 ± 0.20aB	11.86 ± 0.12aC	11.83 ± 0.15aB

Note: Data in the table are mean ± SE. The different small letter followed the data in the same row and capital letters followed in the same column indicate significant difference at P < 0.05, respectively, by Turkey’s multiple comparison.

**Table 4:** ANOVA of effects of transgenic rice plants infected with SRBSDV on nymph survival rate and duration of BPH.

Factors	Nymph survival rate		Nymph duration of the female		Nymph duration of the male (d)	
	F	P	F	P	F	P
SRBSDV Infection	0.038	0.846	0.339	0.564	0.103	0.750
Rice variety (line)	0.719	0.493	18.07	<0.001	39.47	<0.001
SRBSDV Infection *Rice variety (line)	0.108	0.898	5.13	0.010	1.06	0.355

### Effects of transgenic rice plants infected with SRBSDV on the sex ratio and wing differentiation of BPH

Rice variety and SRBSDV infection did not significantly affect the sex ratio of BPH. There was also no significant interaction between rice variety and SRBSDV infection on the sex ratio of BPH or the proportion of brachypterous and macropterous adults (Tables 5 and 6).

**Table 5:** Effects of transgenic rice plants infected with SRBSDV on the sex ratio and wing differentiation of BPH.

Rice	The sex ratio (♀:♂)		wing differentiation			
			Female brachypterous: (brachypterous + macropterous)		Male brachypterous: (brachypterous + macropterous)	
	Un-infected rice plants	Infected by SRBSDV	Un-infected rice plants	Infected by SRBSDV	Un-infected rice plants	Infected by SRBSDV
T1C-19	1.39:1aA	1.18:1aA	0.89:1aA	1.00:1aA	0.49:1aA	0.67:1aA
T2A-1	1.12:1aA	1.00:1aA	0.91:1aA	0.78:1aB	0.49:1aA	0.65:1aA
MH63	1.41:1aA	1.09:1aA	1.00:1aA	0.88:1aAB	0.51:1aA	0.71:1aA

**Table 6:** ANOVA of effects of transgenic rice plants infected with SRBSDV on the sex ratio and wing differentiation of BPH.

Factors	The sex ratio (♀:♂)		Wing differentiation of female		Wing differentiation of male	
	F	P	F	P	F	P
SRBSDV Infection	1.192	0.281	1.285	0.263	6.374	0.016
Rice variety (line)	0.481	0.621	1.978	0.151	0.907	0.907
SRBSDV Infection *Rice variety (line)	0.069	0.933	3.690	0.034	0.033	0.968

### Effects of transgenic rice plants infected with SRBSDV on the body weight, fecundity, and egg hatchability of female adult BPH

The body weight and fecundity of female adult, egg hatchability of BPH feeding on different rice lines were different. There was no significant difference found on the weight and fecundity of female adult BPH being fed on SRBSDV-infected T1C-19 and T2A-1 with those being fed on uninfected plants. Rice varieties and SRBSDV infected plants also had no significant effects on hatching rates of BPH (Table 7).

**Table 7:** Effects of transgenic rice plants infected with SRBSDV on the body weight, fecundity of female adult BPH and egg hatchability.

Rice	Body weight of female (mg/♀)		Fecundity (Eggs/♀)		Egg hatchability (%)	
	Un-infected rice plants	Infected by SRBSDV	Un-infected rice plants	Infected by SRBSDV	Un-infected rice plants	Infected by SRBSDV
T1C-19	2.00 ± 0.05aA	2.02 ± 0.05aA	70.18 ± 10.69aA	69.50 ± 15.34aA	96.89 ± 1.34aA	93.90 ± 1.59aA
T2A-1	1.94 ± 0.05aA	2.00 ± 0.05aA	79.50 ± 12.59aA	55.33 ± 6.63aA	96.38 ± 1.44aA	96.38 ± 2.34aA
MH63	2.05 ± 0.05aA	2.05 ± 0.07aA	62.33 ± 8.08aA	51.63 ± 7.00aA	91.91 ± 3.22aA	94.49 ± 2.34aA

Variance analysis showed no significant interactions between rice variety and SRBSDV infection on the body weight, fecundity of female adults, or egg hatchability (Table 8).

**Table 8:** ANOVA of effects of transgenic rice plants infected with SRBSDV on the body weight, fecundity of female adult BPH and egg hatchability.

Factors	Body weight of female		Fecundity		Egg hatchability	
	F	P	F	P	F	P
SRBSDV Infection	0.975	0.381	1.195	0.313	0.034	0.855
Rice variety (line)	0.061	0.806	0.613	0.438	0.588	0.560
SRBSDV Infection *Rice variety (line)	0.320	0.727	0.815	0.449	0.875	0.424

### Effects of transgenic rice plants infected with SRBSDV on the longevity of adult BPH

There were no significant effects on the longevities of male and female BPH being fed on SRBSDV-infected T1C-19, T2A-1 or MH63 plants. The longevity of male adult BPH on uninfected MH63 plants was however shorter than that of male adults fed on uninfected T1C-19 and T2A-1 plants (Table 9).

**Table 9:** Effects of transgenic rice plants infected with SRBSDV on the longevity of adult BPH.

Rice	Longevity of female (d)		Longevity of male (d)	
	Un-infected rice plants	Infected by SRBSDV	Un-infected rice plants	Infected by SRBSDV
T1C-19	12.07 ± 0.88aA	10.87 ± 0.94aA	13.57 ± 0.34aA	12.40 ± 0.27bA
T2A-1	13.78 ± 1.45aA	12.50 ± 1.71aA	15.22 ± 1.13aA	14.17 ± 1.54aA
MH63	10.80 ± 1.25aA	12.00 ± 1.25aA	10.00 ± 0.91aB	11.50 ± 1.25aA

Variance analysis showed that rice variety and SRBSDV infection did not significantly affect the longevity of female adult BPH and there was no significant interaction between rice variety and SRBSDV infection. The longevity of male adults feeding on uninfected rice lines differed significantly, however there was no significant interaction between SRBSDV infection and rice variety (Table 10).

**Table 10:** ANOVA of effects of transgenic rice plants infected with SRBSDV on the longevity of adult BPH.

Factors	Longevity of female (d)		Longevity of male (d)	
	F	P	F	P
SRBSDV Infection	0.183	0.670	0.120	0.730
Rice variety (line)	1.103	0.339	9.503	<0.001
SRBSDV Infection *Rice variety (line)	0.693	0.504	1.664	0.198

## Discussion

The introduction of the exogenous Bt gene changed some rice traitswhich might affect some species in the community^16^. As use of transgenic pest-resistant crops increase in the future the potential environmental risks of such crops might raise converns^7, 17^. Bt toxins are important insecticidal proteins in Bt crops. The expression level of Bt toxin is a key factor that determines its insecticidal effect. However, the expression level of Bt protein in rice varies at different growth stages and in different parts of the plant. Zhang *et al*.^18^ showed that Cry1Ab content in Huachi B6 rice changed throughout development (grain filling stage > heading stage > mature stage > tillering stage) and varied among organs (panicle > stem or leaf > root). Bt protein expression may also be affected by other factors. Bruns and Abel^19^ found that nitrogen fertilizers increased Bt protein expression in Bt maize. Zhang *et al*.^20^ found that the expression of Bt protein was influenced by nitrogen treatment (medium nitrogen treatment > high nitrogen treatment > nitrogen free treatment) during the cotton leaf development stage, functional stage and senescence stage. The expression level of Bt toxins in cotton variety RCH-134 was increased by 29.43%, 36.02% and 69.37%, respectively, after plants were sprayed three times with imidacloprid^21^. Rice planthoppers continuously ingest Bt protein when they feed on Bt rice, but it is not clear whether the Bt protein affects their ability to transmit the SRBSDV. The results of our experiments showed that the infection rate of SRBSDV was not significantly different among Bt rice lines T1C-19 and T2A-1 and non-transgenic parental rice MH63. Moreover, the Cry protein content in Bt rice lines T2A-1 and T1C-19 did not change significantly after SRBSDV infection. This suggests that the introduction of an exogenous Bt gene into the rice genome will have no significant effect on the infection and epidemic of SRBSDV. SRBSDV infection also has no significant effect on the expression of Bt protein in Bt rice.

The relationship between plant viruses and vector arthropods is complex. Plant viruses rely on mediators to propagate and spread and the virus can directly induce physiological changes in vectors by circulating and replicating in them. In addition, viruses and vectors share the same host plants and compete for plant resources. At the same time, the presence of viruses and vectors can induce defensive reactions in host plants and affect insect growth and behavioral responses indirectly through changes in the content of host plant nutrients and secondary metabolites^22^. Host plants, arthropod vectors and viruses interact closely^23^. In the middle and lower region of the Yangtze River in China, BPH, WBPH and small brown planthopper (SBPH), *Laodelphax striatellus*, share the same host rice plant, but show differences in peak abundance^24^. Feeding on RBSDV-infected rice plants significantly increased the nymphal survival rate of non-vector adult female WBPH, while enhancing their resistance to hunger, increasing their fecundity, and prolonging their longevity^20, 25, 26^. The aarasitoid of rice planthopper *Anagrus nilaparvatae* was found to show an obvious preference for rice plants infected with SRBSDV. The parasitoid rate of BPH eggs was significantly increased in infected rice plants^27^. After the non-vector BPH was fed on SRBSDV infected plants their nymphal survival rates decreased significantly, but the developmental duration, body weight and sex ratio were not significant changed. Female adult BPH developing on SRBSDV infected rice did not show significant changes in longevity or fecundity. The hatchability of BPH eggs on SRBSDV-infected plants was however significantly higher than that of eggs on healthy plants^15^. Infection of rice with SRBSDV had little effect on the feeding behavior of BPH, but the time required for BPH to reach the feeding site was increased^28^. Similar results were obtained in this study, for BPH fed on T1C-19 infected with SRBSDV, exogenous Bt protein expression and SRBSDV infection did not significantly affect the ecological fitness of BPH, except that the developmental duration of females and the longevity of male BPH was significantly shortened in comparison with those of BPH being fed on healthy T1C-19 rice plants. Transgenic Cry1Ac, Cry2AA and Cry1Ca rice lines had no unfavorable effects on fecundity, survival, growth, and physiological processes of digestion, detoxification and immune responses of BPH^29^, Lu *et al*.^28^ reported that the laboratory results showed the development duration of each life stage (apart from fourth-instar nymphs), nymphal survival rate, female fecundity, and the egg hatching rate of BPH being fed on Bt (T1C-19 and T2A-1) were not different from those on non-Bt rice MH63^30^. The field trials further revealed the population density of BPH was not significantly influenced by T1C-19 and T2A-1 rice lines^30^. The longevity of adult BPH being fed on MH63 plants (infected with SRBSDV or not) was slightly shorter than on T2A-1 or T1C-19 plants. This might be due to the slightly lower amino acid content of the MH63 plants.

## Methods

### Insects

BPH and WBPH were continuously maintained in a control chamber at the Zhejiang Academy of Agricultural Sciences (26 ± 1 °C, relative humidity 70–90%, 12-hour light/12-hour dark cycle) on the susceptible rice variety TN1.

### Rice varieties

Transgenic Bt rice lines (T1C-19 and T2A-1) and their non-Bt parental rice MH63 were used in the laboratory test. T1C-19 is a transgenic Bt rice line with Cry1C* gene based on *Cry1Ca5*, and T2A-1 is a transgenic Bt rice line with *Cry2A* gene^31, 32^, and both of them were developed to manage lepidoptera pests in China. MH63 is an elite indica restorer strain for cytoplasmic male-sterile in China and served as the non-transgenic control in this study. All the rice seeds were provided by the National Key Laboratory of Crop Genetic Improvement, Huazhong Agricultural University.

### Obtaining the SRBSDV-infected rice plants

Second instar WBPH nymphs were placed in a beaker with wet filter paper. After 2 hours of starvation, they were transferred to rice plants infected with SRBSDV. After 2 days, they were transferred to healthy rice plants for 7 days. Then ten WBPH carrying SRBSDV were transferred into each beaker with 10 rice seedlings of T1C-19, T2A-1, or MH63 at the 2-leaf stage, respectively, according to the method of Yang *et al*.^33^. WBPH were removed after 2 days, and rice was transplanted into the net chamber. After 30 days in the net chamber, one leaf from each rice plant was collected and molecularly identified following the method of Ji *et al*.^34^. The rates of diseased plants with SRBSDV were used. Healthy rice plants were considered as the control group.

### The susceptibility of different transgenic varieties

Twenty WBPH carrying SRBSDV were transferred into each beaker with 10 rice seedlings of T1C-19, T2A-1, or MH63 at the 2-leaf stage, respectively. WBPH were removed after 2 days. Each treatment was replicated ten times. Thirty days later, one leaf from each rice plant was collected and molecularly identified following the method of Ji *et al*.^19^. The susceptibilities of tested 3 rice lines to SRBSDV infection were calculated.

### Cry protein contents in SRBSDV-infected transgenic rice lines T2A-1 and T1C-19

After virus inoculation, T2A-1 or T1C-19 rice leaves (0.1 g) were collected from the same position on each plant. After grinding with liquid nitrogen, each sample was with extraction reagent. Supernatants were collected by centrifugation, after which Cry protein abundance was measured using an Envirologix kit.

### The effects of SRBSDV-infected rice plants on ecological parameters of BPH

Survival tests used the methods described by He *et al*.^35^. The 45-day-old SRBSDV-infected and healthy rice plants with outer sheathes and inactive roots removed were carefully cleaned with tap water and placed individually in a glass tube (diameter 2.5 cm, height 18 cm) with 1.5 cm deep Kimura B nutrient solution. Ten newly hatched nymphs of BPH were introduced to a prepared test tube plugged with medical purified cotton. Test tubes were then kept in a climate chamber at 26 ± 1 °C, 75–90% relative humidity (RH) and L12: D12 light cycle. Rice plants were replaced regularly and the Kimura B nutrient solution was replenished. Each treatment was replicated for ten times. The numbers of surviving nymphs and adults were recorded daily and newly emerged female adults were weighed individually within 2 h.

For other tests, similar rice plants were prepared and put into a test tube (diameter 2.5 cm, height 30 cm) with 1.5 cm deep in Kimura B nutrient solution. One pair of adults newly emerged in 12 h were introduced into a tube sealed with medical purified cotton. All test tubes were kept in a climate chamber at 26 ± 1 °C, 75–90% RH and L12: D12 light cycle. The survival of adults was recorded daily. Rice plants and Kimura B nutrient solution were replaced regularly. Newly hatched nymphs were counted and removed every day until no nymph was observed for three days. Tillers were dissected for counting un-hatched eggs under the microscope. Each treatment was replicated for ten times.

### Statistical analysis

SPSS 18.0 software was used to analyze the data. Before performing statistical analyses, the percentage data for nymph survival rates and egg hatchability were transformed into arcsine and square root values respectively. Statistical significance was tested by Student’s t-test or single-factor multivariate analysis of variance. Descriptive statistics were expressed as mean ± standard error. The threshold of significance was *P* < 0.05.
